# PianoGym: Safe post-action piano rhythm training with fatigue constraints

**DOI:** 10.1371/journal.pone.0351141

**Published:** 2026-06-16

**Authors:** Xiaoyu Meng, Hui Shi, Ningning Liu, Zhuangzhuang Pan, Yan Xia

**Affiliations:** 1 College of Humanities and Arts, Xi’an International University, Xi’an, China; 2 School of Business, Suzhou Vocational University, Suzhou, Jiangsu, China; 3 Institute for Advanced Studies, Universiti Malaya, Kuala Lumpur,‌‌ Malaysia; 4 Digitization Department, Suzhou University of Technology, Suzhou, Jiangsu, China; Universidad Nacional de Tres de Febrero, ARGENTINA

## Abstract

Bimanual piano rhythm training must maintain precise interlimb timing under limited practice time and under fatigue constraints, while feedback on performance is typically available only after an exercise. A piano practice gym environment (PianoGym) is used as a reproducible simulator for fatigue-constrained piano rhythm training under post-action feedback. The training task is formulated as a fatigue-constrained, post-action, partially observable Markov decision process (POMDP). In this POMDP, a controller observes beat asynchrony, dominance gap, synchronization fidelity, and two fatigue signals, and selects the next exercise from a finite library of structured practice actions. To handle delayed measurements and fatigue feasibility under the simulator budget, we introduce a dual-timescale safety layer. The slow Lagrangian part tracks a long-horizon average constraint using revealed true fatigue, while the fast predictive guard screens candidate actions using the online fatigue estimate. On top of this layer, a piano model predictive controller (PianoMPC) uses certainty-equivalent planning and performs finite-horizon rollouts over a calibrated surrogate environment model and searches only within guard-filtered action sets. In the main three-profile experiment, PianoMPC achieves mean time-to-mastery values of 24.4 to 28.2 steps and FeasibleRate values of 0.90 to 0.95 under the shared environment-side guard. Under the same environment-side guard, it also outperforms bandit and value-based agents. These results indicate that model-predictive planning can convert a fixed operational fatigue budget into faster progress in fatigue-aware piano practice within the PianoGym simulator and its stated surrogate fatigue and skill-dynamics assumptions.

## Introduction

Bimanual piano rhythm training requires precise interlimb timing while operating under fatigue limits and limited practice time [1,2]. In typical instructional settings, the coordination state is inferred only after an exercise, from three music-specific observables, namely beat asynchrony (ms), dominance gap (ms), and synchronization fidelity in [0,1]. The next exercise must be selected before new measurements become available [3,4]. This post-action observation pattern is further complicated by recent findings that synchronization stability depends on effector, modality, and presentation order, and that training trajectories are non-monotonic and context-dependent [5–8]. These results imply that practice should be adapted to measurements rather than follow fixed routines. In this paper, safety refers to fatigue feasibility under an explicitly defined simulator budget, not to clinical or physical safety certification for human learners. Under these conditions, we must design a controller that converts a limited fatigue budget into faster progress while keeping both average and peak fatigue within allowed limits.

Related work can be grouped in three directions. First, music motor research has characterized rhythmic coordination, effector dependence, rate specificity, and variability profiles. However, these results are typically analyzed offline and are seldom converted into online decision rules that operate directly on music-specific observables and current fatigue levels [3,4,7,9–11]. Second, safe and constrained decision making has developed runtime enforcement and Lagrangian-style methods. Yet many controllers still encode safety only through soft penalties or keep evaluation separate from selection, which makes it difficult to reuse the same safety interface during data collection and testing [12–15]. Third, planning and sequencing for music sessions and real-time accompaniment have shown the benefit of goal-aware ordering and model-based synchronization. However, learner-side skill transfer, interference across limbs, and explicit fatigue constraints are often omitted [16–19]. A unified formulation is therefore required to connect structured skill dynamics, post-action observations, and explicit safety constraints in a form that can be deployed in instructional environments.

The present study considers a constrained training problem in which a controller receives post-action observations of asynchrony, dominance gap, and synchronization fidelity. From these observations, it must select the next structured practice action from a finite library. Each action is tagged with difficulty, skill targets, learning and transfer parameters, time cost, and a positive fatigue increment. The setting exhibits delayed measurements and partial observability of multi-skill proficiency. It also features heterogeneous transfer across left hand, right hand, polyrhythm, and switching skills, together with limited exploration due to safety requirements and lesson length. The central challenges are to act on post-action observations, to model transfer, interference, fatigue accumulation, and retention under noise, and to enforce safety on two time scales while preserving progress toward mastery.

To address these challenges, a piano practice gym environment (PianoGym) is defined. PianoGym implements a post-action, fatigue-constrained partially observable Markov decision process (POMDP) with a structured practice library and music-specific observables. A dual-timescale safety layer combines a Lagrangian update that adapts to long-horizon average fatigue with a short-horizon predictive guard that filters actions whose predicted fatigue would violate the safety margin. On top of this layer, piano model predictive control (PianoMPC) rolls out the model over a finite horizon and searches only within the safe action sets returned by the guard. Experiments in the PianoGym environment compare this controller with bandit and value-based agents under the same environment-side guard in order to isolate the effect of planning depth and agent-level safety components. The main contributions of this paper include:

- We formulate fatigue-aware bimanual piano rhythm training as a post-action POMDP that connects structured practice actions, music-specific observables, latent skill dynamics, and fatigue constraints in a single reproducible benchmark.
- We introduce PianoMPC, a certainty-equivalent model-predictive controller that performs fatigue-aware lookahead planning over a finite practice library. This design enables the controller to allocate the same simulator fatigue budget more effectively toward faster mastery.
- We evaluate PianoMPC across three learner profiles and a 3 × 3 task suite under a shared environment-side guard. PianoMPC reduces mean TTM compared with reactive and value-based baselines while maintaining simulator-level fatigue feasibility.

The remainder of the paper is organized as follows. Section Related work reviews related work. Section Methods presents the modeling assumptions, the safety mechanisms, and the PianoMPC controller. Section Experiments describes the task suite, learner profiles, and evaluation metrics, and reports results on efficiency, feasibility, and robustness. Section Conclusion concludes and outlines possible extensions. Section Limitations discusses the scope of the certainty-equivalence, fatigue, and skill-coupling assumptions.

## Related work

### Observables and coordination in piano practice

Music-facing observables obtained from Musical Instrument Digital Interface (MIDI) or sensor systems make it possible to condition practice on measured coordination changes. Studies on effectors, modality, and tempo show that synchronization stability depends on hand, presentation, and rate rather than being uniform across conditions, which motivates interfaces that operate directly on these music-specific measurements [3,4]. Recent piano-specific modeling work shows that skilled piano control cannot be reduced to simple timing accuracy alone. Upper-limb coordination during octave playing depends on biomechanical constraints [20]. Expressive piano output also reflects nonlinear mappings between dynamic control parameters and sound [21]. Brink et al. and Maarup et al. further reported that variability structure and a bodily hierarchy across voice, hands, and feet shape coordination transitions. These findings in turn motivate explicit noise modeling and the use of standardized observables for practice controllers [9,22]. Longitudinal neuroimaging on piano training further indicates context-dependent, non-monotonic plasticity, so progress cannot be treated as stationary during practice [7,23,24].

Fatigue studies on pianists show that repetitive or demanding segments induce local muscular fatigue and degrade timing and key velocity. They also show that subjective rest does not always match objective indicators [1,2]. These findings support returning a noisy online fatigue estimate for immediate action filtering together with a delayed true fatigue value for long timescale statistics. Interpersonal and duet studies report that joint action and perturbations modulate interbrain synchronization and the balance of self other integration [11,25]. Such effects are outside the scope of the single-learner, post-action interface considered here and are therefore not modeled.

### Safety in sequential decision making

Safe and constrained reinforcement learning encodes performance under cost limits through Lagrangian or proximal updates, which yields policies that track average constraints during learning [14,26]. Complementary lines of work enforce safety through certified sets and predictive shielding that override unsafe actions near constraint boundaries and provide forward-invariance style guarantees [15,27]. More recent studies combine runtime enforcement with learned policies in safety-critical systems, where an external enforcer corrects an agent whenever formal safety rules would be violated [12,28–30].

A large fraction of these approaches assumes fully observed states or synchronous cost feedback and operates on a single time scale, as in representative value-based reinforcement learning under full observability [31,32]. Shielding under partial observability is closely related because it uses a model-based safety filter when the agent state is incomplete [33]. In fatigue-aware piano rhythm training the controller receives post-action music metrics and two distinct fatigue signals. Safety must therefore be split into a slow Lagrangian adaptation that uses revealed true fatigue and a fast predictive guard that uses the online estimate. Experiments in PianoGym are designed with a shared environment-side guard so that different decision rules can be compared under the same fatigue budget.

### Planning and model predictive controller for practice

Optimization based sequencing on music platforms shows that exploiting position aware and locally sequential preferences increases within session engagement. This supports ordered practice rather than independent ranking of exercises [18]. Model based real time accompaniment further demonstrates that temporal alignment with human performers improves when the controller adapts online [19]. These studies validate goal aware, model based lookahead in musical interaction. However, they generally do not model learner side skill transfer, interference across tasks, or fatigue limited practice budgets, and the control inputs are typically not drawn from a finite library.

Model predictive controllers have been adapted for rhythmic or periodic motor tasks. Examples include basis function parameterization for fast gait generation and combinations of central pattern generators with MPC [34,35]. Gain scheduled and bio rhythm informed MPC maintains performance under time varying operating points, which indicates that predictive controllers can respect physiological cycles without losing responsiveness [36,37]. The piano practice setting considered here differs because actions come from a finite, structured library with tagged difficulty, skill targets, and positive fatigue increments, and planning is restricted to guard-filtered safe action sets. The combination of post-action observability, dual-timescale safety, and library-based MPC is not addressed in the above MPC and sequencing literature.

## Methods

### Problem statement and interaction protocol

Fatigue-aware piano practice is modeled as a constrained, post-action, partially observable Markov decision process (CPOMDP) with music-specific observables and two fatigue signals. This common interface makes it possible to analyze both sample efficiency and safety.

### Post-action partially observable process

At decision step *t*, the learner occupies an unobserved latent state


$$\begin{array}{c} s_{t}=({𝐱}_{t},f_{t},{𝐦}_{t})\in [0,1{]}^{K}\times [0,1]\times [0,1{]}^{K} \end{array}$$ (1)

where **x**_*t*_ contains the proficiencies of *K* rhythm-related skills, *f*_*t*_ is the accumulated cognitive and motor fatigue, and **m**_*t*_ is a memory or retention trace of the same dimension.

The controller maintains an internal estimate ${\hat{s}}_{t}$ and chooses a structured practice action according to


$$\begin{array}{c} a_{t}\sim \pi (\cdot |{\hat{s}}_{t}),\;a_{t}\in 𝒜 \end{array}$$ (2)

**Fig 1:**
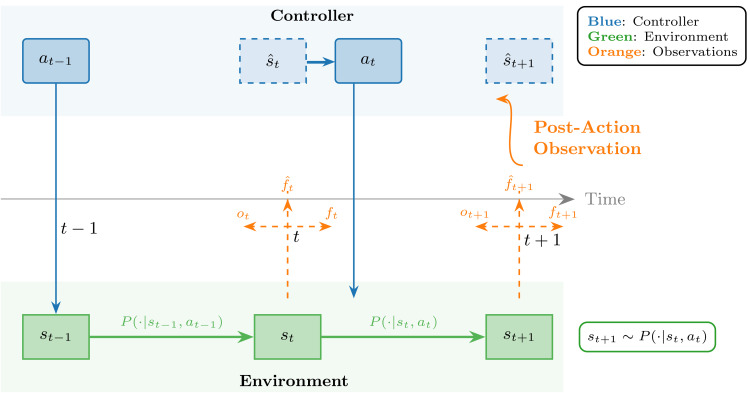
Post-action interaction protocol.

The environment then evolves according to the true state


$$\begin{array}{c} s_{t+1}\sim P(\cdot |s_{t},a_{t}) \end{array}$$ (3)

that is, the transition kernel depends on (*s*_*t*_, *a*_*t*_) and not on the estimate. After the transition, the environment reveals three post-action signals


$$\begin{array}{c} s_{t}\overset{a_{t}\;}{\to }s_{t+1}\overset{\text{reveal}\;}{\to }(o_{t+1},{\hat{f}}_{t+1},f_{t+1}),\;r_{t}=ℛ(o_{t+1},a_{t}) \end{array}$$ (4)

where


$$\begin{array}{c} o_{t+1}=({\text{Async}}_{t+1},{\text{DomGap}}_{t+1},{\text{Fid}}_{t+1}) \end{array}$$ (5)

collects the music-facing measurements, ${\hat{f}}_{t+1}\in [0,1]$ is a noisy online fatigue estimate intended for immediate safety screening, and $f_{t+1}\in [0,1]$ is the revealed true fatigue intended for long-run constraint adaptation. The scalar *r*_*t*_ denotes the immediate reward revealed after executing *a*_*t*_, and ℛ(·) is the reward map from the post-action observation and executed action to this scalar reward. The two fatigue signals are consumed by different safety components in Section Dual-timescale fatigue safety. Policies in Section PianoMPC controller are defined on the filtered state estimate ${\hat{s}}_{t}$, as shown in Fig 1.

### Objective and constraints

Rhythm mastery is declared once


$$\begin{array}{c} \text{Async}\leq 60\text{ms},\;|\text{DomGap}|\leq 20\text{ms},\;\text{Fid}\geq 0.8 \end{array}$$ (6)

hold for *W* = 3 consecutive decision steps, where *W* denotes the consecutive-mastery window. The main performance indicator is the time to mastery (TTM), defined as the smallest index *t* at which Eq. (6) is satisfied, with $\text{TTM}=T_{\max}$ if the condition is not met within the episode.

Because every practice segment induces fatigue, the process is constrained by the long-run average of the true fatigue:


$$\begin{array}{c} C_{f}(\pi )={𝔼}_{\pi }[\frac{1}{T}\sum_{t=1}^{T}f_{t}]\leq \tau \end{array}$$ (7)

with task-dependent threshold τ∈(0,1), where $T\leq T_{\max}$ denotes the episode length in the PianoGym environment. The control objective is therefore


$$\begin{array}{c} \max_{\pi }\;{𝔼}_{\pi }[\sum_{t=1}^{T}r_{t}]\;\text{s.t.}\;C_{f}(\pi )\leq \tau \end{array}$$ (8)

Here $C_{f}(\pi )$ denotes the expected long-run fatigue cost under policy π, ${𝔼}_{\pi }$ is expectation over trajectories induced by π and the simulator dynamics. In the PianoGym instantiation used for experimentation, the cumulative reward in Eq. (8) is monotone in TTM, so reporting TTM is sufficient to compare controllers. The reward-based formulation is nevertheless retained because the model-predictive controller in Section PianoMPC controller optimizes a finite-horizon surrogate of Eq. (8).

### Environment and predictive model

The controller performs model-predictive rollouts and therefore requires a deterministic approximation of the environment dynamics. This section makes the abstract CPOMDP in Section Problem statement and interaction protocol concrete by specifying the latent variables, the structured action library, the skill and fatigue updates, and the stochastic observation layer from which rewards are computed. All parameters below are treated as known constants of the PianoGym environment and are not learned by the controller. This makes PianoMPC a certainty-equivalent planner: it plans from the current filtered estimate as if that estimate were the rollout state, rather than maintaining a full posterior belief over latent skills.

### Parameterization and calibration

Parameterization and calibration PianoGym uses a fixed parameterization so that all controllers are compared in the same simulator rather than learning a different environment model. The action-library structure, skill targets, transfer signs, and fatigue-load ordering were manually specified from the musical roles of the exercises. The numerical learning rates, forgetting rate, fatigue gain, rest recovery rate, observation scales, and noise levels were then set once on an internal calibration suite of simulated pilot profiles spanning balanced, mild-left-weak, and severe-left-weak learners. The calibration target was not to fit a human cohort, but to place asynchrony, dominance gap, fidelity, fatigue, and TTM in interpretable ranges aligned with the evaluation thresholds. After this calibration step, the parameters were frozen for every baseline, ablation, and robustness experiment. Sensitivity to these choices is assessed in the dynamics-mismatch, scoped-mismatch, threshold-window, and guard-dependence analyses in Section Experiments. Table 1 summarizes these parameter groups and their sources.

**Table 1:** Parameter sources in PianoGym.

Group	Examples	Source	Used
Design constants	exercise library, skill targets, REST	manual design from exercise roles	yes
Calibration constants	learning rates, observation scales, noise	simulated pilot profiles	yes
Sensitivity-tested constants	fatigue gain, forgetting, gating sharpness	calibrated values varied in ablations	yes

### State and action space instantiation

The latent skill vector contains *K* = 5 rhythm-relevant components


$$\begin{array}{c} {𝐱}_{t}=[x_{L,t},x_{R,t},x_{2:3,t},x_{3:4,t},x_{\text{switch},t}{]}^{⊤} \end{array}$$ (9)

describing left-hand control, right-hand control, 2:3 polyrhythm coordination, 3:4 polyrhythm coordination, and fast switching. The memory trace ${𝐦}_{t}\in [0,1{]}^{K}$ has the same dimension and plays the role of a smoothed retention level.

The action set 𝒜 is a finite library of predefined structured practice actions and is exposed to the controller as its discrete action space. Each action is encoded as


$$\begin{array}{c} a=(d_{a},{𝐪}_{a},\alpha_{a},{𝐌}_{a},\beta_{a},c_{a}) \end{array}$$ (10)

where $d_{a}\in [0,1]$ is a difficulty tag, ${𝐪}_{a}\in {\mathbb{R}}_{+}^{K}$ with $\|{𝐪}_{a}{\|}_{1}=1$ specifies which skills the segment targets, $\alpha_{a}\in {\mathbb{R}}_{+}^{K}$ specifies learning rates per skill, ${𝐌}_{a}\in {\mathbb{R}}^{K\times K}$ is a sparse transfer or interference matrix, $\beta_{a}\in [0,\beta_{\max}]$ is the positive fatigue load, and $c_{a}\geq 0$ is the execution time cost. This representation separates the musical target, the learning intensity, and the physiological load. The library also contains an explicit REST action,


$$\begin{array}{c} {𝐪}_{\text{REST}}=0,\;\alpha_{\text{REST}}=0,\;{𝐌}_{\text{REST}}=0,\;\beta_{\text{REST}}=0 \end{array}$$ (11)

which is kept available at every decision step.

### Skill dynamics

**Fig 2:**
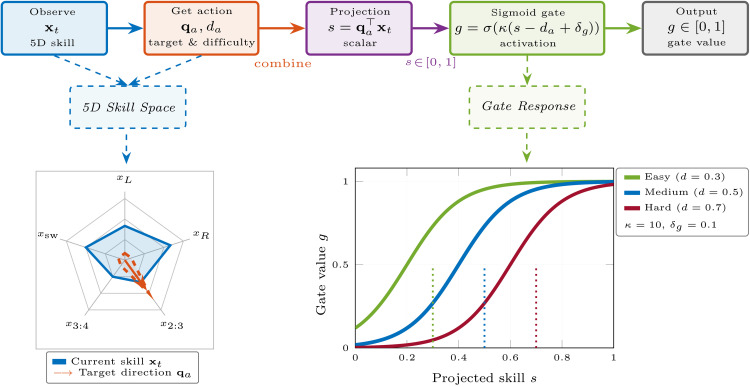
Difficulty-matching gate mechanism.


Skill adaptation ${𝐱}_{t}\to {𝐱}_{t+1}$ results from diminishing returns, difficulty matching, and fatigue attenuation. For an executed action *a*_*t*_,


$$\begin{array}{c} \Delta {𝐱}_{t}^{\text{learn}}=(\alpha_{a_{t}}⊙(1-{𝐱}_{t}))⊙g({𝐪}_{a_{t}},{𝐱}_{t},d_{a_{t}})\cdot h(f_{t}) \end{array}$$ (12)

where $\left(1-{𝐱}_{t}\right)$ reduces gains near mastery. The difficulty matching gate, as shown in Fig 2, is used to filter candidate actions so that their tagged difficulty stays close to the current skill estimate.

$$\begin{array}{c} g(𝐪,𝐱,d)=\sigma (\kappa ({𝐪}^{⊤}𝐱-d+\delta_{g})),\;\sigma (z)=\frac{1}{1+e^{-z}} \end{array}$$ (13)

which becomes active when the projected skill level ${𝐪}^{⊤}𝐱$ approaches the declared difficulty *d*.

Fatigue attenuates learning through


$$\begin{array}{c} h(f_{t})=\max\{\underset{―}{h},1-\gamma_{f}f_{t}\} \end{array}$$ (14)

with rate $\gamma_{f}>0$ and lower bound $\underset{―}{h}\in (0,1)$.

Transfer, interference, and process noise are then added:


$$\begin{array}{c} \Delta {𝐱}_{t}=\Delta {𝐱}_{t}^{\text{learn}}+{𝐌}_{a_{t}}{𝐪}_{a_{t}}+\epsilon_{t},\;\epsilon_{t}\sim 𝒩(0,\sigma_{x}^{2}𝐈) \end{array}$$ (15)

The next skill vector is clipped element-wise to the admissible range


$$\begin{array}{c} {𝐱}_{t+1}=\mathrm{clip}({𝐱}_{t}+\Delta {𝐱}_{t},0,1) \end{array}$$ (16)

where, for a vector *v*, clip(v,0,1) applies $\min\{1,\max\{0,v_{i}\}\}$ to every coordinate *v*_*i*_. This clipping is part of the environment step and is mirrored by the controller.

### Fatigue and memory dynamics

For practice actions $a_{t}\neq \text{REST}$, fatigue increases with the action load:


$$\begin{array}{c} f_{t+1}=\mathrm{clip}(f_{t}+\beta_{a_{t}},0,1),\;0\leq \beta_{a_{t}}\leq \beta_{\max} \end{array}$$ (17)

For the REST action, fatigue recovers at rate ρ>0:


$$\begin{array}{c} f_{t+1}=\mathrm{clip}(f_{t}-\rho ,0,1) \end{array}$$ (18)

Retention is maintained through an exponential smoother:


$$\begin{array}{c} {𝐦}_{t+1}=\lambda {𝐦}_{t}+(1-\lambda ){𝐱}_{t+1},\;\lambda \in (0,1) \end{array}$$ (19)

and forgetting is represented as a small decay proportional to missing retention:


$$\begin{array}{c} {𝐱}_{t+1}\leftarrow {𝐱}_{t+1}-\eta_{\text{forget}}(1-{𝐦}_{t+1})⊙{𝐱}_{t+1} \end{array}$$ (20)

with decay rate $\eta_{\text{forget}}$. This decay is also part of the dynamics mirrored by the controller.

### Observation and reward generation

Given $s_{t+1}=({𝐱}_{t+1},f_{t+1},{𝐦}_{t+1})$ and the executed action *a*_*t*_, the environment samples


$$\begin{array}{c} o_{t+1}=({\text{Async}}_{t+1},{\text{DomGap}}_{t+1},{\text{Fid}}_{t+1}) \end{array}$$

from explicit action-dependent observation equations. Let **w**_*A*_ be the fixed skill weights used for asynchrony, let $\phi_{a}$ and $\psi_{a}$ be action-specific scale parameters, and let $\xi_{a}$ be an action-specific dominance-gap offset.


$$\begin{array}{cc} \mu_{t+1}^{A} & =\phi_{a_{t}}(1-{𝐰}_{A}^{⊤}{𝐱}_{t+1})(1+0.3f_{t+1}),\\ {\text{Async}}_{t+1} & =\max\{0,\mu_{t+1}^{A}+\epsilon_{t+1}^{A}\} \end{array}$$ (21)

where $\epsilon_{t+1}^{A}\sim 𝒩(0,\sigma_{A}^{2})$. The dominance gap is generated as


$$\begin{array}{cc} \mu_{t+1}^{D} & =\psi_{a_{t}}(x_{R,t+1}-x_{L,t+1})+\xi_{a_{t}},\\ {\text{DomGap}}_{t+1} & =\mu_{t+1}^{D}+\epsilon_{t+1}^{D} \end{array}$$ (22)

where $\epsilon_{t+1}^{D}\sim 𝒩(0,\sigma_{D}^{2})$. The fidelity signal is a clipped logistic-normal proxy


$$\begin{array}{cc} \ell_{t+1} & =\eta_{0}+\eta_{1}{𝐪}_{a_{t}}^{⊤}{𝐱}_{t+1}-\eta_{2}d_{a_{t}}-\eta_{3}f_{t+1},\\ {\text{Fid}}_{t+1} & =\mathrm{clip}(\sigma (\ell_{t+1})+\epsilon_{t+1}^{F},0,1),\;\epsilon_{t+1}^{F}\sim 𝒩(0,\sigma_{F}^{2}) \end{array}$$ (23)

These equations make the quantitative link explicit: higher relevant skill lowers asynchrony and improves fidelity, left-right imbalance changes dominance gap, and higher fatigue increases timing error and lowers fidelity. They are simulator observation equations, not fitted physiological measurement laws. The online fatigue estimate is obtained by


$$\begin{array}{c} {\hat{f}}_{t+1}=\mathrm{clip}(f_{t+1}+\epsilon_{f},0,1),\;\epsilon_{f}\sim 𝒩(0,\sigma_{f}^{2}) \end{array}$$ (24)

To aggregate heterogeneous observables into a single learning signal, each measurement *v* is standardized using fixed statistics $\left(\mu_{v},\sigma_{v}\right)$:


$$\begin{array}{c} z(v)=\frac{v-\mu_{v}}{\sigma_{v}},\;z(v{)}^{+}=\max\{0,z(v)\} \end{array}$$ (25)

Here *z*(*v*)^+^ denotes the positive part of the standardized residual. Asynchrony and dominance gap are standardized in this way. Fidelity already lies in [0,1] and is used in raw form. The immediate reward is


$$\begin{array}{c} r_{t}=-[w_{1}z({\text{Async}}_{t+1}{)}^{+}+w_{2}|z({\text{DomGap}}_{t+1})|+w_{3}(1-{\text{Fid}}_{t+1})]-\lambda_{c}c_{a_{t}} \end{array}$$ (26)

where $w_{1},w_{2},w_{3}\geq 0$ are fixed reward weights, $c_{a_{t}}$ is the duration cost of the executed action, and $\lambda_{c}$ is its coefficient. The reward penalizes poor musical coordination and long practice segments and keeps duration-related effects comparable across actions.

### Dual-timescale fatigue safety

**Fig 3:**
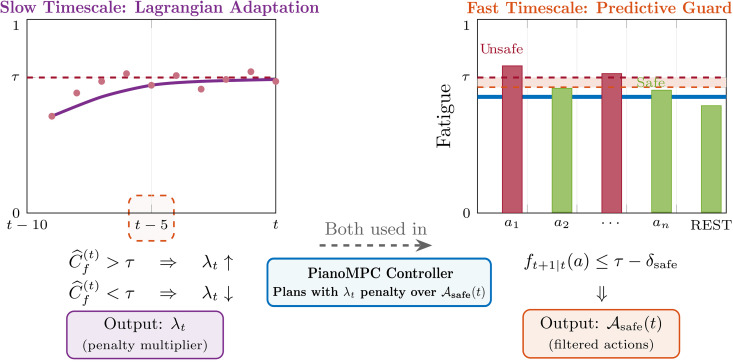
Dual-timescale safety layer.

Fatigue must remain within specified limits both on average and at the next decision instant. Safety is therefore enforced on two coupled time scales, as shown in Fig 3. A slow Lagrangian adaptation tracks the long-run constraint in Eq. (7) using the revealed true fatigue. A fast predictive guard filters unsafe actions using the online estimate. In this paper, the safety layer refers to the simulator-level fatigue-feasibility interface used to screen practice actions. During experimentation, the same one-step guard is implemented as an environment-side guard for all agents in order to provide a common safety interface. The environment-side guard takes precedence and can overwrite any action proposed by a controller according to a fixed fallback rule.

### Slow timescale: lagrangian adaptation

At the beginning of step *t*, the controller has access to the true fatigue *f*_*t*_ that was revealed at the end of the previous transition. An exponential moving average of revealed fatigue is updated as


$$\begin{array}{c} {\hat{C}}_{f}^{\left(t\right)}=(1-\eta_{C}){\hat{C}}_{f}^{\left(t-1\right)}+\eta_{C}f_{t} \end{array}$$ (27)

and the Lagrange multiplier for fatigue is advanced by projected gradient ascent


$$\begin{array}{c} \lambda_{t}=\max\left\{0,\lambda_{t-1}+\eta_{\lambda }({\hat{C}}_{f}^{\left(t\right)}-\tau )\right\} \end{array}$$ (28)

The maximum operator keeps the Lagrange multiplier nonnegative, as required for the fatigue inequality constraint. The scalar $\lambda_{t}$ converts the average constraint into an instantaneous penalty and is kept fixed for the entire decision step *t*. During planning in Section PianoMPC controller, this multiplier penalizes predicted trajectories whose fatigue rises above τ.

### Fast timescale: predictive guard

The fast component restricts actions that would produce an immediate overload. At step *t*, the internal fatigue estimate ${\hat{f}}_{t}$ (computed in Section PianoMPC controller) is combined with the deterministic dynamics Eq. (17)–Eq. (18) and the action-specific loads $\beta_{a}$ in order to predict the next-step fatigue $f_{t+1|t}(a)$ for every a∈𝒜. Two margins are used for two different purposes. The conservative one-step margin $\epsilon_{\text{step}}$ defines a primary step cap, whereas the relaxed guard slack $\delta_{\text{guard}}$ defines a peak diagnostic threshold used by the environment-side guard and by guard-consistency experiments. The primary one-step set is


$$\begin{array}{c} {𝒜}_{\text{step}}(t)=\{a\in 𝒜:f_{t+1|t}(a)\leq \tau -\epsilon_{\text{step}}\} \end{array}$$ (29)

For a guard horizon *H*_*g*_, the relaxed peak condition is


$$\begin{array}{c} \max_{1\leq h\leq H_{g}}f_{t+h|t}(a)\leq \tau +\delta_{\text{guard}} \end{array}$$ (30)

The executed guard uses Eq. (29) as the ordinary action filter, and REST is always available. The relaxed band in Eq. (30) is logged for calibration and can affect execution only in the fallback case where no non-rest primary action survives; otherwise it does not enlarge the ordinary safe set. Accordingly, $\tau -\epsilon_{\text{step}}$ is used as the conservative execution cap, whereas $\tau +\delta_{\text{guard}}$ is used only as a relaxed peak-diagnostic threshold. When the environment-side guard is present, the environment applies the same interface to the finally proposed action.

### PianoMPC controller

This section introduces PianoMPC, a finite-horizon, certainty-equivalent controller that plans only over actions certified as safe by the dual-timescale mechanism. The controller uses the deterministic model in Section Environment and predictive model, the multipliers in Section Dual-timescale fatigue safety, and the same structured action library as the environment.

### Certainty-equivalent state tracking

At the start of step *t*, immediately after receiving $\left(o_{t},{\hat{f}}_{t},f_{t}\right)$ generated by action $a_{t-1}$, the controller forms an updated estimate


$$\begin{array}{c} {\hat{s}}_{t}=({\hat{𝐱}}_{t},{\hat{f}}_{t},{\hat{𝐦}}_{t}) \end{array}$$

The update is carried out in two stages.

First, a prediction step propagates the previous estimate through the deterministic model:


$$\begin{array}{c} \left({\hat{𝐱}}_{t|t-1},{\hat{f}}_{t|t-1},{\hat{𝐦}}_{t|t-1})=F({\hat{s}}_{t-1},a_{t-1}\right) \end{array}$$ (31)

where F(·) applies the noise-free dynamics Eq. (12)–Eq. (20) and Eq. (17)–Eq. (18). This produces a prior for step *t*.

Second, a correction step aligns the fatigue component with the revealed true fatigue while keeping the skill and memory components unchanged:


$$\begin{array}{c} {\hat{𝐱}}_{t}={\hat{𝐱}}_{t|t-1} \end{array}$$ (32)

$$\begin{array}{c} {\hat{𝐦}}_{t}={\hat{𝐦}}_{t|t-1} \end{array}$$ (33)

$$\begin{array}{c} {\hat{f}}_{t}=(1-\eta_{f})\;{\hat{f}}_{t|t-1}+\eta_{f}f_{t} \end{array}$$ (34)

This certainty-equivalent design is motivated by two characteristics of the setting. Musical observables (Async, DomGap, Fid) are noisy and task dependent, so back-propagating them into all skill coordinates at every step would increase variance and computational load. In contrast, the environment reveals the true fatigue without noise. Using it to anchor ${\hat{f}}_{t}$ yields an accurate fatigue signal for both the slow Lagrangian update and the fast guard. The observables are therefore used through the deterministic observation and reward model in Section Environment and predictive model and are not used for state correction.

### Finite-horizon planning

**Fig 4:**
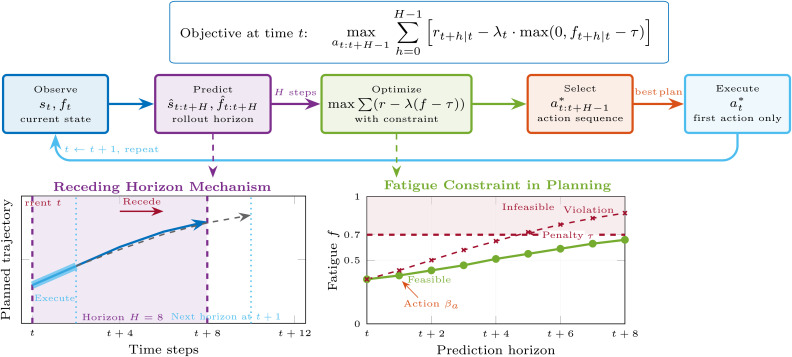
Model predictive control with receding horizon.


Given ${\hat{s}}_{t}$ and the current multiplier $\lambda_{t}$, the controller solves a receding-horizon problem of length *H*, as shown in Fig 4. Let a candidate action sequence be $a_{t:t+H-1}$. The deterministic model is rolled forward as

$$\begin{array}{c} {\hat{s}}_{t+h+1|t}=F({\hat{s}}_{t+h|t},a_{t+h}),\;h=0,\ldots ,H-1 \end{array}$$ (35)

starting from ${\hat{s}}_{t|t}={\hat{s}}_{t}$. At each lookahead step *t* + *h*, the predictive guard in Section Dual-timescale fatigue safety is applied to produce the future safe set ${𝒜}_{\text{safe}}(t+h)$ using the predicted fatigue contained in ${\hat{s}}_{t+h|t}$.

The immediate reward at lookahead index *h* is obtained by applying the deterministic observation-and-reward model to the predicted state ${\hat{s}}_{t+h+1|t}$ and to action $a_{t+h}$. For compactness, this predicted quantity is denoted by $r_{t+h|t}$. The planning problem is


$$\begin{array}{c} \max_{a_{t:t+H-1}}\;\sum_{h=0}^{H-1}[r_{t+h|t}-\lambda_{t}(f_{t+h|t}-\tau )] \end{array}$$ (36)

$$\begin{array}{c} \text{s.t.}\;{\hat{s}}_{t+h+1|t}=F({\hat{s}}_{t+h|t},a_{t+h}) \end{array}$$ (37)

$$\begin{array}{c} a_{t+h}\in {𝒜}_{\text{safe}}(t+h),\;h=0,\ldots ,H-1 \end{array}$$ (38)

The multiplier $\lambda_{t}$ computed in Eq. (28) is held constant over the horizon, so that the optimization in Eq. (36) depends only on the predicted trajectories.

The discrete optimization in Eq. (36) is solved by a beam search of width *b*. At each depth, only the best *b* partial sequences consistent with the safe sets are kept, which yields a computational cost proportional to b·H and to the size of the filtered action sets. After evaluating all depth-*H* sequences retained by the beam, the first action of the best sequence is executed. At the next decision step, the state is re-estimated and the planning procedure is repeated. This produces a receding horizon controller that respects both long-run and short-horizon fatigue constraints and that is used in Section Experiments.

### Experiments

This section evaluates whether model-predictive planning improves learning under fatigue-constrained practice. All experiments use the same PianoGym action library and the same environment-side short-horizon safety guard. Two safety regimes are reported. In the main comparison reported in Section Main comparison under the safety guard, the environment fatigue hard constraint is enabled for the three learner profiles, so that agents differ only in their decision rules under a common safety interface. In the task suite study reported in Section Robustness across task families, each profile follows its default configuration. The balanced and mild-left-weak profiles keep only the guard, whereas the severe-left-weak profile enables both the guard and the hard constraint.

## Dataset

### Task composition

A 3 × 3 task suite is constructed in PianoGymEnv (see Table 2). The rows are the three learner profiles used throughout the experiments, and for each profile three transfer levels (weak, medium, strong) are used. This gives 9 tasks in total.

**Table 2:** Profiles in the PianoGym task suite.

Profile	Dynamics	Environment hard constraint	Remark
Balanced	stationary	disabled	symmetric skill dimensions
Mild-left-weak	non-stationary	disabled	mild lateral asymmetry
Severe-left-weak	non-stationary	enabled	tighter fatigue threshold τ

### Scale and execution protocol

Unless otherwise stated, *S* = 10 independent random seeds are run for each task, and each seed generates one trajectory. A trajectory runs for at most $T_{\max}$ steps and terminates early once the mastery condition is satisfied. This produces


$$\begin{array}{c} 9\times S\times T_{\max},\;T_{\max}=1024 \end{array}$$

records of states, actions, observations, and fatigue. Under this configuration the total sample size is on the order of 10^5^, which is sufficient for reporting means and 95% confidence intervals.

### Symmetry between left-weak and right-weak profiles

Only the left-weak variants (mild-left-weak, severe-left-weak) are reported in the main text. The data generator can swap left and right skill dimensions and action targets to obtain the right-weak counterparts. Because the action-library fatigue parameters, the short-horizon safety guard, and the PianoMPC state estimator are symmetric in the two dimensions, the mirrored tasks yield the same rankings. Their safety statistics are also almost identical. The corresponding results can be reproduced from the released code or from the supplementary material.

### Observation and action interface

The environment uses a post-action interface. After executing action *a*_*t*_ at time *t*, it returns


$$\begin{array}{c} o_{t+1}=({\text{Async}}_{t+1},{\text{DomGap}}_{t+1},{\text{Fid}}_{t+1}) \end{array}$$

together with the online fatigue estimate ${\hat{f}}_{t+1}$ and the true fatigue $f_{t+1}$. The action set 𝒜 contains several structured practice actions and an explicit rest action REST. For comparability, a common environment-side short-horizon safety guard is applied to every agent. Each agent first proposes a candidate action. The guard then filters actions using the current online fatigue estimate ${\hat{f}}_{t}$ (denoted $f_{t|t}$ in the experiments) and produces


$$\begin{array}{c} {𝒜}_{\text{step}}(t)=\{\;a\in 𝒜|{\hat{f}}_{t}+\beta_{a}\leq \tau -\epsilon_{\text{step}}\;\} \end{array}$$

with $\epsilon_{\text{step}}=0.05$ in the default experiments. The environment-side guard also logs whether the proposed action would pass the relaxed diagnostic band $\tau +\delta_{\text{guard}}$, with $\delta_{\text{guard}}=0.08$. Only actions in this primary set are executed, except for the relaxed fallback case defined in Section Dual-timescale fatigue safety. Some agents, such as PianoMPC and the fatigue-aware LinUCB variant, forecast future fatigue and therefore tend to propose actions that already satisfy the guard. Other agents do not anticipate the guard, but their final executed actions are still filtered by the same environment-side rule. This design keeps the safety mechanism identical across methods and lets the comparison focus on the quality of the decision rules.

### Experimental setup

One simulation step corresponds to a practice segment of about 30–60 seconds. This correspondence fixes the scale of the fatigue load $\beta_{a}$, the recovery rate ρ, and the typical time to mastery (TTM). All observation scalings, such as $\phi_{a}$, $\psi_{a}$, $\xi_{a}$, and associated noise levels are estimated on an internal calibration set and then frozen for all experiments. The calibration set contains simulated pilot runs from the three learner profiles and was used only to set observation ranges and noise levels before the reported experiments were run.

Unless specified otherwise, the experiments use *K* = 5 skill dimensions and an action library of size |𝒜|=12, which includes the REST action. The mastery condition requires a window of *W* = 3 consecutive successful steps. Key hyperparameters are set as follows: the profile-specific fatigue threshold is τ∈{0.75,0.70,0.65} for balanced, mild-left-weak, and severe-left-weak profiles; the one-step guard margin is $\epsilon_{\text{step}}=0.05$; the relaxed diagnostic slack is $\delta_{\text{guard}}=0.08$; and the guard horizon is $H_{g}=1$. For the main comparison, the MPC planning horizon is *H* = 3; the horizon sweep separately evaluates H∈{1,3,5,10}. The beam search width is *b* = 20. Dynamics parameters include a memory smoothing factor λ=0.95 and a forgetting rate $\eta_{\text{forget}}=0.02$. The reward weights are set to $\left(w_{1},w_{2},w_{3})=(0.4,0.2,0.4\right)$, and the time-cost coefficient is $\lambda_{c}=0.01$.

The per-step computational complexity of PianoMPC is given by $O(b\cdot H\cdot |{𝒜}_{\text{safe}}(t)|\cdot T_{F})$, where *T*_*F*_ is the cost of one forward prediction F(·). In practice, the finite action library combined with the predictive guard ensures that the safe action set $\left|{𝒜}_{\text{safe}}(t)\right|$ remains small, keeping the controller computationally efficient.

### Evaluation metrics

All results are computed by the evaluation module in PianoGym and are averaged across tasks and seeds. Unless otherwise stated, means and 95% confidence intervals are computed across independent seeds within the stated profile or task aggregation unit. For rate metrics, figure displays are clipped to the valid interval [0,1] or [0,100]% without changing the underlying means. Consider a trajectory


$$\begin{array}{c} \{(o_{t},a_{t},r_{t},f_{t}){\}}_{t=1}^{T} \end{array}$$

where $f_{t}\in [0,1]$ denotes fatigue, τ denotes the environment fatigue threshold, *W* denotes the mastery window, and $T_{\max}$ denotes the maximum episode length.

Time to mastery (TTM) quantifies sample efficiency by measuring how many steps are required to achieve stable mastery for a window of length *W*. Formally,


$$\begin{array}{c} \text{TTM}=\min\{\;t|mastery\_count(t)\geq W\;\} \end{array}$$ (39)

with $\text{TTM}=T_{\max}$ if the mastery condition is not satisfied within $T_{\max}$. TTM is the primary performance metric used to rank agents in Section Main comparison under the safety guard to Section Effect of fatigue-threshold and mastery-window choices (lower is better). The counter mastery_count(t) records consecutive successful steps up to and including step *t* and resets to zero whenever any criterion in Eq. (6) is not satisfied.

Task return is computed by recording the instantaneous raw reward $r_{t}^{raw}$ and aggregating


$$\begin{array}{c} \text{TotalReward}=\sum_{t=1}^{T}r_{t}^{raw} \end{array}$$ (40)

$$\begin{array}{c} {\overset{―}{r}}^{raw}=\frac{1}{T}\sum_{t=1}^{T}r_{t}^{raw} \end{array}$$ (41)

Safety metrics follow the dual-timescale safety design, consisting of a short-horizon guard and a long-horizon fatigue limit. Each fatigue sample is thresholded as


$$\begin{array}{c} v_{t}=\max(0,f_{t}-\tau ) \end{array}$$

Following standard practice in constrained and safe reinforcement learning [14], the following quantities are reported:


$$\begin{array}{c} \text{AvgFatigue}=\frac{1}{T}\sum_{t=1}^{T}f_{t} \end{array}$$ (42)

$$\begin{array}{c} \text{AvgViolation}=\frac{1}{T}\sum_{t=1}^{T}v_{t} \end{array}$$ (43)

$$\begin{array}{c} \text{OverloadRate}=\frac{1}{T}\sum_{t=1}^{T}1[v_{t}>0] \end{array}$$ (44)

$$\begin{array}{c} \text{FeasibleRate}=1-\text{OverloadRate} \end{array}$$ (45)

FeasibleRate and OverloadRate are complementary by definition, while AvgViolation measures the magnitude of fatigue violations rather than their frequency. Guard replacement rate is reported as an intervention diagnostic for proposed-versus-executed actions and is not treated as a safety success metric. For this reason, the results are interpreted as fatigue-feasibility outcomes under the simulator budget rather than as absolute safe or unsafe behavior.

A progress metric called rhythm independence gain is also reported:


$$\begin{array}{c} \text{IndependenceGain}=\frac{1}{3}[({\text{async}}^{init}-{\text{async}}^{final})+(|\text{dom}{|}^{init}-|\text{dom}{|}^{final})+({\text{fid}}^{final}-{\text{fid}}^{init})] \end{array}$$ (46)

This metric uses the same thresholds as the mastery condition defined by the environment and therefore supports cross task comparison. Additional diagnostics, including memory retention and offline evaluation coverage, appear in the public implementation.

## Main comparison under the safety guard

### Baselines

All baselines observe the same post-action signals, use the same action set, and are filtered by the same environment-side guard. Our PianoMPC agent and the fatigue-aware LinUCB variant also run the same short-horizon guard inside the policy, whereas the other baselines rely only on the environment-side guard. Thus differences in Section Main comparison under the safety guard come mainly from the decision rule. Thompson [38] is a lightweight posterior-sampling bandit under delayed feedback. LinUCB [39] is a contextual bandit on PianoGym features but does not plan future fatigue. Bayesian MAB [40] adds discounting and change-point handling for non-stationary profiles. CCB-DF [41] learns from delayed counterfactual rewards. DQN [31] is a value-based agent that relies on the external guard for safety. Safe-AC [32] treats fatigue above τ as cost in a Lagrangian actor-critic. Auto-Curriculum [42] orders exercises and inserts REST, mimicking human teaching without MPC-style planning.

### Results on three profiles

This section reports the main comparison under the common safety guard. Table 3 summarizes three learner profiles and eight agents. FeasibleRate values range from 0.89 to 0.97 in the main comparison, with nonzero overload rates still possible. For this reason, the primary comparison is mean TTM interpreted together with FeasibleRate and violation diagnostics, rather than a binary safe/unsafe label.

**Table 3:** Overall comparison of time to mastery (TTM) and FeasibleRate (Feas.). Values are mean ± 95% CI over 10 runs. Best values are highlighted in bold and second best are underlined.

	Balanced		Mild-left-weakness		Severe-left-weakness	
Agent	TTM ↓	Feas. ↑	TTM ↓	Feas. ↑	TTM ↓	Feas. ↑
Thompson	43.2 ± 5.7	0.94 ± 0.02	46.4 ± 11.8	0.92 ± 0.03	45.3 ± 8.0	0.90 ± 0.03
DQN	56.6 ± 15.7	0.90 ± 0.02	45.0 ± 13.9	0.91 ± 0.03	50.7 ± 10.1	0.91 ± 0.03
LinUCB	47.7 ± 7.0	**0.97 ± 0.02**	52.0 ± 9.0	**0.96 ± 0.02**	48.0 ± 6.6	**0.96 ± 0.03**
AutoCurriculum	51.8 ± 9.1	0.93 ± 0.01	59.4 ± 14.8	0.94 ± 0.03	59.7 ± 11.0	0.94 ± 0.02
BayesianMAB	35.1 ± 7.0	0.89 ± 0.03	40.1 ± 5.4	0.90 ± 0.04	39.9 ± 6.4	0.93 ± 0.04
Safe-AC	43.3 ± 5.2	0.91 ± 0.04	43.0 ± 3.9	0.92 ± 0.02	41.2 ± 4.4	0.92 ± 0.04
CCB-DF	41.6 ± 6.4	0.94 ± 0.02	47.0 ± 5.2	0.93 ± 0.02	49.8 ± 7.3	0.93 ± 0.02
PianoMPC	**24.4 ± 3.6**	0.95 ± 0.02	**26.0 ± 5.6**	0.91 ± 0.03	**28.2 ± 3.3**	0.90 ± 0.04

PianoMPC achieves mean TTM values of 24.4, 26.0, and 28.2 steps across the balanced, mild-left-weak, and severe-left-weak profiles, respectively. This range is clearly below the second group, formed by BayesianMAB with 35–40 steps. All other methods require between the low 40s and about 60 steps, with LinUCB, DQN, CCB-DF and AutoCurriculum often around 50–60 steps.

**Fig 5:**
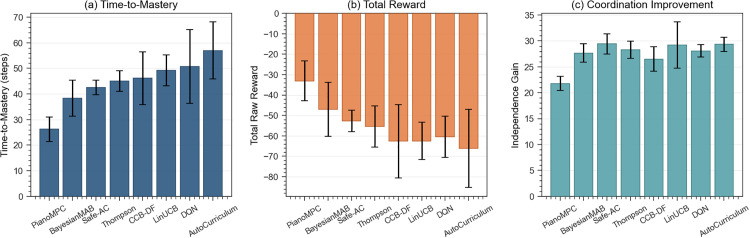
Quantitative results for the main experiment. Panel labels show mean TTM, reward, and coordination gain; error bars are 95% seed CIs.

**Fig 6:**
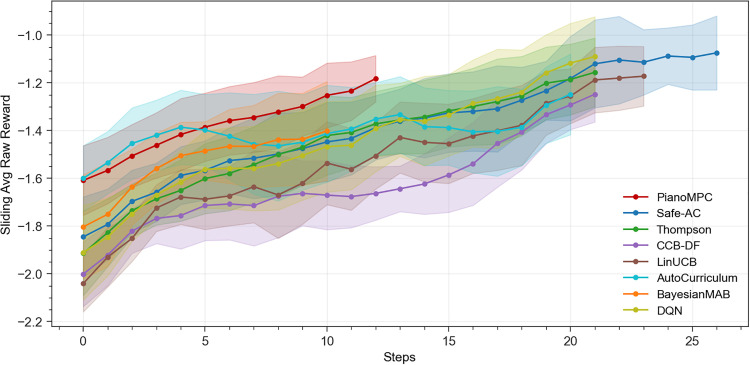
Learning curves for the main experiment. Curves show sliding-averaged raw reward; shaded bands are 95% seed CIs.

Fig 5 panel (a) shows the same three-level structure. LinUCB keeps the highest FeasibleRate, about two points above PianoMPC, yet this improvement is marginal compared with the extra training time. The large gap appears because LinUCB reacts myopically when fatigue is near the limit and spends many steps on low-value rest choices. The controller plans the fatigue budget over the horizon, so it converts the same fatigue allowance into progress. Fig 5 panel (b) follows the time pattern, since shorter runs accumulate less negative reward. Panel (c) reports that some slower methods reach slightly higher coordination, which reflects longer exposure rather than stronger learning rules. Fig 6 shows that PianoMPC improves reward earliest and keeps the advantage through the early practice steps. This behaviour is consistent with planning under post-action observations and with the dual safety design of the environment. A guard-dependence ablation compares the default environment-side guard, a weaker guard, and no guard. PianoMPC remains the fastest method in all three settings, with macro mean TTM values of 25.67, 24.50, and 25.77 steps and FeasibleRate values of 0.921, 0.920, and 0.927, respectively. Under the default environment-side guard, the guard replacement rate for PianoMPC is 0.000, whereas replacement rates for several reactive baselines are much higher. This indicates that the guard is a common execution interface and diagnostic, rather than the sole driver of PianoMPC’s efficiency.

### Effect of planning horizon

**Fig 7:**
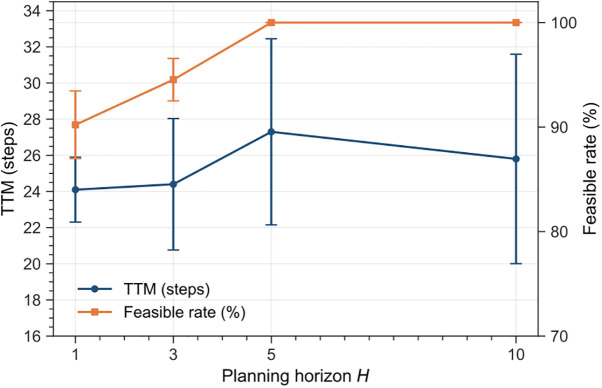
Effect of PianoMPC planning horizon on the balanced profile. Left axis shows TTM; right axis shows FeasibleRate on a zoomed scale with visual headroom above 100%; symmetric 95% CIs are clipped to the valid 0–100% range for display.

To assess how much lookahead is needed, the planning horizon *H* was varied. This test used the balanced profile while all other settings were fixed. The sweep uses the same balanced-profile seed schedule and agent-update pipeline as the main comparison, so the *H* = 3 point matches the PianoMPC entry in Table 3; *H* = 10 is an additional sweep setting rather than the table configuration. Fig 7 shows the resulting trade-off between training speed and fatigue feasibility. A myopic policy (*H* = 1) achieves the lowest balanced-profile mean TTM at 24.1 steps, but its FeasibleRate is 0.902 and its macro FeasibleRate across profiles is 0.850. The *H* = 1 setting is therefore treated as a low-feasibility setting rather than categorically unsafe.

At the main-comparison setting *H* = 3, the balanced-profile mean TTM is 24.4 steps and FeasibleRate is 0.945. Longer horizons raise FeasibleRate to 1.000 for *H* = 5 and *H* = 10, with balanced-profile mean TTM values of 27.3 and 25.8 steps. This pattern indicates that short lookahead already captures useful near-term fatigue structure, while longer lookahead mainly changes the feasibility-speed trade-off. Thus planning depth, not only the shared guard, explains the efficiency gap.

### Effect of agent-level safety components

**Fig 8:**
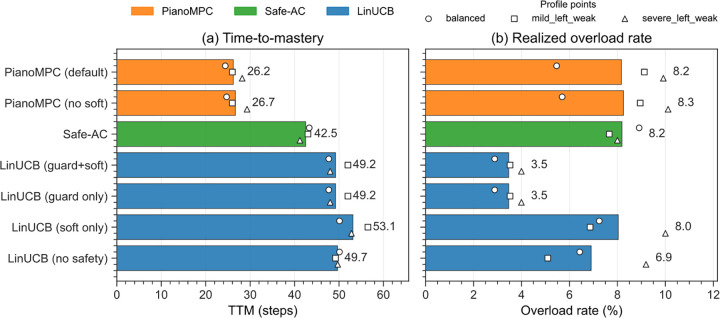
Agent-side safety-module ablation across three learner profiles. Bars show macro means; open markers show profile-specific values. Overload rate denotes the realized trajectory overload rate, 100×(1−FeasibleRate), after the shared environment-side guard.

This experiment evaluates the safety modules inside the agent policy. The short-horizon guard and the soft fatigue penalty are enabled or removed. Fig 8 reports a profile-aware bar plot of mean TTM and realized fatigue-overload rate, computed as 100×(1−FeasibleRate), across all three learner profiles. Note that the environment guard remains enabled for every variant and the ablation removes only agent-side components such as the internal guard or the soft penalty.

With both PianoMPC safety components active, the macro mean realized overload rate is 8.2% and mean TTM is 26.2 steps. Removing the soft penalty gives a similar realized overload rate of 8.3% and a mean TTM of 26.7 steps. Thus, under the shared environment-side guard, PianoMPC’s internal guard and replanning dominate this ablation, while the soft penalty has only a small effect on the realized feasibility-speed trade-off.

The LinUCB variants remain slower, with mean TTM values between 49.2 and 53.1 steps. Their realized overload rates stay between 3.5% and 8.0%, indicating a clearer guard-dependent feasibility-speed trade-off for the reactive bandit variants. Safe-AC appears between these groups, with mean TTM of 42.5 steps and an 8.2% realized overload rate, so it improves over the bandit variants but does not match PianoMPC’s efficiency.

### Consistency between windowed and peak-guard safety metrics

**Fig 9:**
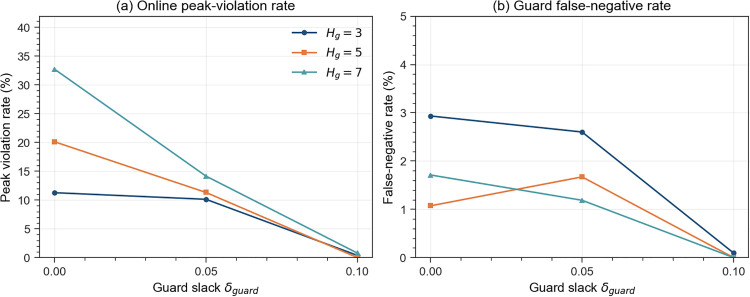
Guard-calibration diagnostics for PianoMPC on the balanced profile. Panels show peak-violation and false-negative rates across guard horizons at fixed diagnostic window $W_{diag}=5$.

This experiment checks whether the online guard diagnostics behave consistently as the guard slack changes. Fig 9 reports online peak-violation rate and guard false-negative rate for PianoMPC on the balanced profile. The diagnostic window is fixed at $W_{diag}=5$ in this plot so that the figure focuses on the guard horizon *H*_*g*_ and the relaxed slack $\delta_{\text{guard}}$.

The online peak-violation rate decreases as $\delta_{\text{guard}}$ increases, which is expected because the diagnostic threshold becomes less conservative. The false-negative rate remains low across the displayed settings and approaches zero at $\delta_{\text{guard}}=0.10$. These rates are diagnostics of the guard calibration; they do not mean that violations are allowed as a goal of the method. The online guard is used for action filtering, whereas windowed violation metrics are retained as offline checks of the realized trajectory.

### Robustness across task families

This experiment tests whether the advantage of planning holds under different task structures. The suite combines three categories (Balanced, Mild-left-weak, Severe-left-weakness) with three transfer levels (weak, medium, strong). Table 4 reports time to mastery and FeasibleRate for the four strongest agents. The suite includes nonzero overload rates in several severe-left-weakness settings, so these values should be read as fatigue-feasibility diagnostics rather than as binary safety claims. We keep these defaults to test robustness under realistic per-task settings rather than under a single globally tuned constraint.

**Table 4:** Robustness of top agents across the 3 × 3 task suite. Cells report mean TTM and FeasibleRate with 95% seed CIs.

Agent	Profile	weak		medium		strong	
		TTM ↓	Feas. ↑	TTM ↓	Feas. ↑	TTM ↓	Feas. ↑
PianoMPC	Balanced	43.0 ± 3.1	1.00 ± 0.00	25.1 ± 3.9	1.00 ± 0.00	13.6 ± 2.8	1.00 ± 0.00
	Mild-left-weak	46.6 ± 3.9	1.00 ± 0.00	30.3 ± 6.7	1.00 ± 0.00	16.5 ± 2.5	1.00 ± 0.00
	Severe-left-weakness	46.3 ± 4.2	0.88 ± 0.04	27.8 ± 1.9	0.91 ± 0.03	17.0 ± 1.3	0.92 ± 0.03
CCB-DF	Balanced	77.7 ± 8.4	1.00 ± 0.00	41.6 ± 7.3	1.00 ± 0.00	34.7 ± 3.7	1.00 ± 0.00
	Mild-left-weak	77.5 ± 7.3	1.00 ± 0.00	45.0 ± 5.6	1.00 ± 0.00	33.2 ± 8.4	1.00 ± 0.00
	Severe-left-weakness	79.9 ± 7.0	0.93 ± 0.02	45.5 ± 5.7	0.90 ± 0.03	28.5 ± 3.3	0.90 ± 0.03
BayesianMAB	Balanced	49.2 ± 7.3	1.00 ± 0.00	26.7 ± 4.2	1.00 ± 0.00	23.1 ± 5.3	1.00 ± 0.00
	Mild-left-weak	54.0 ± 5.2	1.00 ± 0.00	34.4 ± 7.3	1.00 ± 0.00	21.4 ± 4.8	1.00 ± 0.00
	Severe-left-weakness	54.0 ± 7.2	0.95 ± 0.02	36.2 ± 3.8	0.92 ± 0.03	30.3 ± 6.3	0.92 ± 0.04
Thompson	Balanced	71.2 ± 7.8	1.00 ± 0.00	39.6 ± 4.3	1.00 ± 0.00	30.5 ± 5.8	1.00 ± 0.00
	Mild-left-weak	76.3 ± 9.2	1.00 ± 0.00	42.7 ± 4.3	1.00 ± 0.00	24.9 ± 4.7	1.00 ± 0.00
	Severe-left-weakness	75.5 ± 7.2	0.90 ± 0.02	47.6 ± 7.1	0.90 ± 0.03	28.3 ± 6.6	0.89 ± 0.03

PianoMPC achieves the lowest mean TTM in every cell of the table. On weak transfer tasks it needs about 43–47 steps, while CCB-DF needs around 78 steps and Thompson stays above 70 steps. BayesianMAB lies between them, at 49–54 steps, and is therefore the second tier. This shows that, when transfer is scarce and fatigue is enforced, planning still converts practice into progress more efficiently than reactive bandits.

When transfer becomes medium or strong, every agent improves, but the gains are not equal. PianoMPC drops to roughly 25 steps for medium transfer and to 14–17 steps for strong transfer across all categories. BayesianMAB and CCB-DF also speed up, yet they remain 10–20 steps slower than the planner. In the severe-left-weakness tasks, PianoMPC is the only agent that stays below 30 mean steps at medium transfer while keeping FeasibleRate above 0.9. This indicates that the planning controller remains effective across this task suite under the evaluated fatigue limits.

### Robustness to dynamics misspecification

**Fig 10:**
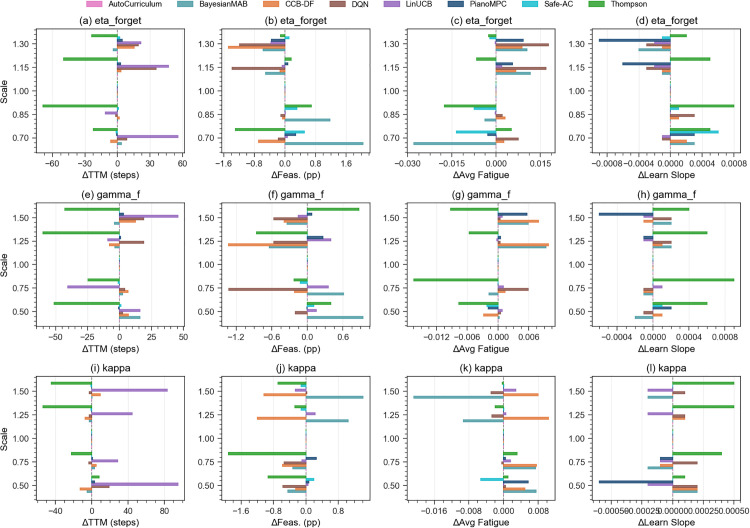
Sensitivity of agents to dynamics misspecification. Panels show metric changes from nominal; AutoCurriculum reaches the run limit in scanned settings.

This experiment studies how sensitive the agents are to errors in the task dynamics. Three environment parameters are scaled around the nominal value one. The parameters control memory decay, fatigue accumulation, and gating sharpness. Fig 10 reports the change in four metrics with respect to the baseline run. Values close to zero indicate stable behaviour under misspecification.

Across all settings, PianoMPC stays close to the zero line. For memory errors, its time to mastery changes by at most about four steps, and FeasibleRate varies within roughly three tenths of a percentage point. Average fatigue and learning slope also show only small shifts. This pattern suggests that online replanning absorbs moderate model errors without extra tuning.

Non-planning agents react much more strongly. For misspecified forgetting rates, LinUCB is affected the most and can require more than 50 additional steps, while AutoCurriculum always hits the run limit in terms of TTM, so its sensitivity in time to mastery cannot be observed. Several bandit baselines also lose around one percentage point of feasibility when the fatigue gain is inaccurate. Misspecification of the gating parameter pushes CCB-DF and LinUCB even further from the origin. These results indicate that methods with fixed exploration schedules are more sensitive to the tested dynamics errors, whereas the predictive controller changes less under the same simulator changes. The zero sensitivity of AutoCurriculum in some panels is therefore a ceiling artifact: it reaches the run limit in the scanned settings, rather than showing greater robustness. Tests with scoped simulator changes also keep the calibrated fatigue model fixed while increasing observation noise and varying transfer/interference strength. In additional scoped simulator-change tests, PianoMPC retained the lowest overall mean TTM and a competitive FeasibleRate when observation noise was increased by two to three times and transfer strength was scaled by 1.2. These results support robustness within the tested simulator changes, but they do not imply robustness to arbitrary model error.

### Effect of fatigue-threshold and mastery-window choices

**Fig 11:**
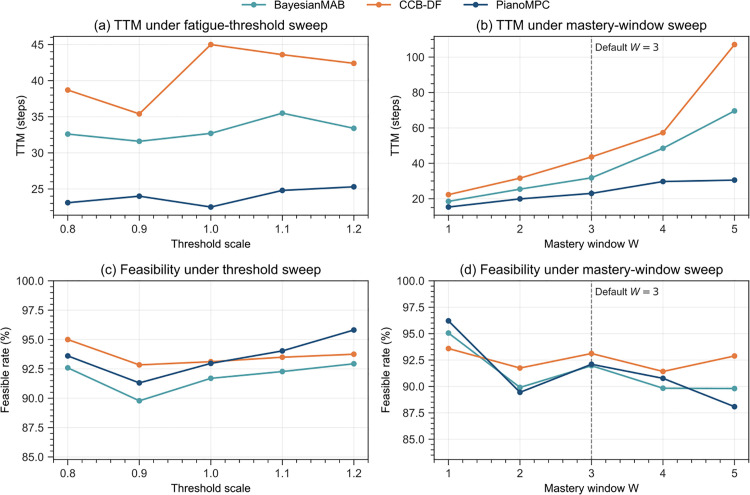
Effect of changing evaluation hyperparameters. Panels show TTM and FeasibleRate under threshold scaling and mastery windows.

The experiment studies how evaluation hyperparameters affect reported performance. Only the fatigue threshold and the mastery window change, agent policies remain identical. Fig 11 reports both TTM and FeasibleRate, directly addressing whether the ranking depends on a single operational mastery or fatigue definition. It also compares PianoMPC, BayesianMAB, and CCB-DF on the balanced profile.

Panel (a) scales the fatigue limit from 0.8 to 1.2 of the nominal value. The PianoMPC curve stays between 22.5 and 25.5 steps. CCB-DF varies from about 35–45 steps and peaks exactly at the unscaled threshold. BayesianMAB moves inside the 31–36 step band and never overtakes PianoMPC. The corresponding FeasibleRate panel shows that the threshold sweep changes feasibility levels but does not remove PianoMPC’s TTM advantage. Thus the relative ordering in Section Main comparison under the safety guard is not caused by a single threshold choice.

The mastery-window sweep is limited to W=1,…,5 because longer windows act mainly as stress tests and can obscure the operational definition of TTM. The default *W* = 3 is marked in both mastery-window panels. PianoMPC remains the fastest method over the displayed window range, while FeasibleRate varies because changing *W* changes episode termination times and the fatigue samples included in the trajectory average. This behaviour shows that TTM is an operational PianoGym metric, not a universal definition of musical mastery.

## Discussion

In PianoGym, model-predictive planning improves mean TTM while maintaining fatigue-feasibility diagnostics under the stated surrogate fatigue model. Under a shared environment-side guard, PianoMPC achieves clearly lower time-to-mastery than reactive bandits. This advantage is supported by the horizon and policy-side safety ablations. The two safety diagnostics are consistent once their purposes are separated. The effect persists across task families, tested dynamics changes, and evaluation hyperparameters. The no-guard and guard-calibration analyses further indicate that PianoMPC’s efficiency is not driven solely by the default environment-side guard. Safety-related results are therefore interpreted as simulator-level fatigue-feasibility findings rather than as claims about real human ‌‌physiology.

## Conclusion

This paper studied sample-efficient learning for fatigue-constrained piano practice under a unified safety interface. PianoGym provides a reproducible simulation benchmark for post-action, fatigue-constrained piano rhythm training, and PianoMPC demonstrates how model-predictive planning can improve mean TTM under this benchmark. Across three learner profiles and the 3 × 3 task suite, PianoMPC achieved the lowest mean TTM while maintaining the reported FeasibleRate range under the evaluated fatigue budget. Ablations on planning depth and agent-level penalties suggest that short lookahead can be sufficient in this simulator and that soft penalties deliver controllable feasibility and speed trade-offs. Alternative safety diagnostics show that tuning the online guard changes absolute rates without reversing the comparative pattern in the evaluated settings.

Robustness tests show stable behavior under the evaluated dynamics misspecification, scoped simulator changes, guard settings, and mastery definitions. The observed advantage was therefore not limited to the default setting in the main table. The resulting claim is simulator-level: PianoMPC is an effective planner for the stated PianoGym surrogate model and fatigue interface. Open directions include learning guard thresholds, richer state estimation, multi-task scheduling, physical-instrument evaluation, and personalized nonlinear fatigue models.

## Limitations

This study has several limitations related to its modeling assumptions. PianoMPC uses a certainty-equivalent design and rolls out a deterministic model from the current filtered estimate. It does not propagate a full posterior over latent skills, fatigue, or observation noise. This choice makes planning efficient for a finite exercise library, but it can understate uncertainty when observations are noisy or learner dynamics change abruptly. PianoGym also represents bimanual rhythm ability with five task-level skill coordinates. These coordinates are coupled by transfer and interference terms. This representation supports controlled benchmarking, but it does not capture the full neuromuscular, biomechanical, or expressive coupling involved in real piano performance.

The scope of the results is also limited by the simulator-level fatigue and evaluation definitions. Fatigue is modeled as a scalar operational index with additive exercise load, linear rest recovery, and a multiplicative effect on learning gain. It is not a validated physiological model of muscular, cognitive, or biomechanical fatigue. Therefore, TTM, FeasibleRate, and mastery thresholds should be interpreted as operational benchmark metrics rather than universal definitions of musical mastery or clinical safety.
